# Identification of the Needs and Preferences of Patients With Cancer for the Development of a Clinic App: Qualitative Study

**DOI:** 10.2196/40891

**Published:** 2023-07-27

**Authors:** Joachim Weis, Lucy Raphaela Wolf, Melanie Boerries, Daniela Kassahn, Martin Boeker, Carolin Dresch

**Affiliations:** 1 Chair for Self-Help Research Comprehensive Cancer Center, Medical Faculty University Clinic Freiburg Freiburg Germany; 2 Institut für Medizinische Bioinformatik und Systemmedizin Medical Faculty University Freiburg University Clinic Freiburg Freiburg Germany; 3 Institute of Artificial Intelligence and Informatics in Medicine Medical Center rechts der Isar, School of Medicine Technical University of Munich Munich Germany; 4 University of Education Freiburg Freiburg Germany

**Keywords:** cancer, mobile app, mHealth, mobile health, needs assessment, patient-centered care, PROM, patient-reported outcome measures, qualitative methods

## Abstract

**Background:**

Mobile health (mHealth) tools were developed during the past decades and are increasingly used by patients in cancer care too. Scientific research in the development of mHealth services is required in order to meet the various needs of patients and test usability.

**Objective:**

The aim of this study is to assess patients’ needs, preferences, and usability of an app (My University Clinic [MUC] app) developed by the Comprehensive Cancer Center Freiburg (CCCF) Germany.

**Methods:**

Based on a qualitative cross-sectional approach, we conducted semistructured interviews with patients with cancer, addressing their needs, preferences, and usability of the designed MUC app. Patients treated by the CCCF were recruited based on a purposive sampling technique focusing on age, sex, cancer diagnoses, and treatment setting (inpatient, outpatient). Data analysis followed the qualitative content analysis according to Kuckartz and was performed using computer-assisted software (MAXQDA).

**Results:**

For the interviews, 17 patients with cancer were selected, covering a broad range of sampling parameters. The results showed that patients expect benefits in terms of improved information about the disease and communication with the clinic staff. Demands for additional features were identified (eg, a list of contact persons and medication management). The most important concerns referred to data security and the potential restriction of personal contacts with health care professionals of the clinical departments of the CCCF. In addition, some features for improving the design of the MUC app with respect to usability or for inclusion of interacting tools were suggested by the patients.

**Conclusions:**

The results of this qualitative study were discussed within the multidisciplinary team and the MUC app providers. Patients’ perspectives and needs will be included in further development of the MUC app. There will be a second study phase in which patients will receive a test version of the MUC app and will be asked about their experiences with it.

**Trial Registration:**

Deutsches Register Klinischer Studien DRKS00022162; https://drks.de/search/de/trial/DRKS00022162

## Introduction

There is a need for more patient empowerment, self-management, and patient participation in health care. Mobile health (mHealth) has proven effective as a technology addressing this need [1]. mHealth is defined by the Global Observatory for eHealth as a medical and public health practice supported by mobile devices, such as mobile phones, patient-monitoring devices, personal digital assistants, and other wireless devices [2]. There is a growing number of mHealth interventions, such as smartphone apps for patients with cancer [3-6]. The range of apps used in oncology is extensive and includes various features, such as symptom assessment through online questionnaires applying patient-reported outcome measures (PROMs), appointment coordination, recommendations for self-care (eg, nutrition, exercise, wound care), and psychological-related self-care (eg, coping). In addition, there are app features, such as diagnosis-specific medical information, medication reminders, access to personal medical data, and social support through interactive communication with peers [4,7-9]. The willingness of patients to use these apps ranges from 52% to 87% [10-12]. However, there are also typical concerns that discourage patients from using such apps: the desire for personal contact with the treating physician, concerns about the security of personal data, and the insecurity about one’s own technical abilities [13-15]*.* In studies, a young age, the male gender, solid technical know-how, a higher socioeconomic status, and higher educational and income levels were associated factors influencing the willingness of patients to use apps in cancer care [10,13,16,17].

As only a small part of mHealth interventions is scientifically evaluated [18], there is a need for including scientific evaluation into the development of mHealth tools already in early phases. Therefore, it is necessary to involve patients in the development process of mHealth apps [19,20] to meet patients' needs for more empowerment and self-management and to develop best-practice features and services for clinical application. However, this recommendation to involve patients at the beginning of the app development process has been rarely followed. This can lead to a lower usage rate due to a lack of a needs-based approach.

Against the background of this study, the Medical Center – University of Freiburg (Germany) developed an app (My University Clinic [MUC]) as a communication tool for patients to support comprehensive cancer care at a large Comprehensive Cancer Center Freiburg (CCCF). The MUC app is not designed as a digital health app but as an information and communication tool for patients at the university clinic. The MUC app includes the following basic functions: (1) appointment management and navigation, (2) access to medical reports, (3) online forms and PROM questionnaires, (4) a health diary to track the development of cancer symptoms and treatment side effects, (5) and general information about the clinic and the disease.

## Methods

### Study Design

The overall aim of our study is to actively involve patients in the development process of the MUC app in order to assess their needs and preferences and investigate their acceptance and usability of the basic structure of the MUC app. The detailed objectives of this qualitative study are (1) to assess the needs, wishes, and preferences of patients with cancer related to the MUC app and (2) to identify patients’ barriers and fears that may limit the use of mHealth and the usability of this patient group. The findings will be incorporated into the app development process, which should help achieve higher acceptance and a higher rate of use. For this purpose, we chose a qualitative study approach and conducted semistructured interviews with patients with cancer using the qualitative content analysis model [21,22] as an explorative approach.

### Recruitment

The inclusion criteria for participation were any cancer diagnosis, current or past cancer treatment at the Medical Center – University of Freiburg, a minimum age of 18 years, and command of the German language. The recruitment period lasted from October 2020 to March 2021. Based on purposive sampling, we distributed information material on our interview study within the Medical Center – University of Freiburg. In addition, we contacted physicians from different departments to address patients. During the recruitment period, we monitored the sampling parameters, focusing on age, sex, cancer diagnoses, and treatment setting (inpatient, outpatient). We consecutively included 17 patients from various oncological departments of the Medical Center – University of Freiburg.

### Data Collection

After conducting a literature search, identifying important issues for patients concerning the needs, barriers, and feasibility related to health apps, a team of multidisciplinary experts (n=4), including physicians, psychologists, biologists, and computer scientists, developed a semistructured interview guide in a multistage consensus process. The final interview guide (see Multimedia Appendix 1) was structured into 13 thematic domains with detailed subqueries. Before the semistructured interviews were conducted and digitally recorded, the patients were introduced to the concept of the MUC app to support cancer care via standardized instructions, including a presentation of the 5 intended main functions of the app, and they completed a questionnaire on demographics and cancer status. All the main functions were presented in an illustrative way. We started the interview asking for general attitudes in terms of app use in daily life and in the health area, followed by an assessment of needs, concerns, and perceived advantages of an app to support cancer care. At the beginning of each domain, the interviewers started with key story–generating questions and optional subqueries [23]. In the second part, the interviewers explained the planned MUC app with its 5 basic features. Patients commented on the basic features and answered questions on how acceptance and usability could be improved. The interviewees got an opportunity to make further suggestions for the MUC app's functions. All interviews were conducted by LRW, author of this paper. The interviews were consecutively transcribed and analyzed to obtain a first overview about the main content categories. Referring to the concept of saturation, we stopped recruitment after verifying that no new aspects emerged from the interview data.

Patients were interviewed in person (n=7, 41.2%) or over the phone due to the COVID-19 pandemic (n=10, 58.8%). The interviews lasted on average 71 minutes (range 60-98 minutes).

### Analysis

The recorded interviews were transcribed verbatim and anonymized. Two scientists with an MSc (authors LRW and CD) coded and analyzed the transcripts independently with the software tool MAXQDA 2020 using content-structuring qualitative content analysis according to Kuckartz [21] and thereby following the model of qualitative content analysis [21,22] to identify themes and subthemes. We combined a deductive and an inductive approach. We formed 13 main categories based on the structure of the interview guide (deductive). Following the inductive approach, we identified subcategories from the interview material. The inductive process was already developed in parallel with the data collection phase, so it was possible to get an idea of whether theoretical saturation (no significantly new topic areas are identified) had been achieved. During this process, the 2 coders discussed the resulting category system until they finally agreed on the final category system. Anchor citations were assigned to the codes, as well as the respective number of people endorsing the code. Using the final hierarchical category system, 12 of 17 (70.6%) interviews were then recoded in a second run to determine the interrater reliability. The kappa (κ_n_) coefficient according to Brennan and Prediger [24] was calculated. The resulting κ_n_=0.93 corresponded to good agreement between coders [25,26].

### Ethical Considerations

Before the start of the study, an ethics vote was obtained from the Ethics Committee of the University in Freiburg (no. 435/20) and the study was registered in the Deutsches Register Klinischer Studien (DRKS; reg. no. DRKS00022162). Before the interviews started, all participants were informed about the study. Participants were included after they provided informed consent. A signed informed consent form was available for all participants. In the transcription of the interviews, we confirm that all patient identifiers were removed or disguised, so the patients described are not identifiable (they cannot be identified through the details of the paper), and the interviews were analyzed anonymously. There was no financial compensation for participation.

## Results

### Description of the Sample

A total of 17 patients (n=8, 47.1%, female and n=9, 52.9%, male) with cancer participated in the needs assessment interviews. As can be seen in Table 1, the patients ranged in age from 26 to 76 years, with a mean age of 54 (SD 13) years. The educational level of the sample was heterogeneous, with most of the patients (n=12, 70.8%) indicating secondary school as their highest school diploma. The sample was heterogeneous in terms of diagnosis, treatment, and tumor status (see Table 2). The patients were in different phases of their cancer treatment. The time since the first diagnosis ranged from 4 months to 16 years (mean 3.6 years, SD 52 months). The most common diagnoses were breast cancer (n=5, 29.4%) and lymphoma (n=3, 17.6%). At the time of the interviews, the majority of patients (n=14, 82.4%) were under ongoing treatment.

**Table 1:** Sociodemographic data of the sample (N=17).

Sociodemographics		Value
**Age (years)**		
	Mean (SD)	54 (13)
	Range	26-76
**Gender, n (%)**		
	Female	8 (47.1)
	Male	9 (52.9)
**Highest education level, n (%)**		
	University	2 (11.8)
	A level	3 (17.7)
	Secondary school	12 (70.8)
**Profession, n (%)**		
	Employee	5 (29.4)
	Pensioner	7 (41.2)
	Self-employed	3 (17.6)
	Unemployed	1 (5.9)
	Civil servant	1 (5.9)

**Table 2:** Medical data of the sample (N=17).

Cancer status		Value
**Diagnosis, n (%)**		
	Breast cancer	5 (29.4)
	Lymphoma	3 (17.6)
	Lung cancer	2 (11.8)
	Brain tumor	2 (11.8)
	Laryngeal cancer	1 (5.9)
	Pancreatic cancer	1 (5.9)
	Skin cancer	1 (5.9)
	Myeloma	1 (5.9)
	Ovarian cancer	1 (5.9)
**Metastasis, n (%)**		
	Yes	3 (17.6)
	No	14 (82.4)
**Disease status, n (%)**		
	Complete remission (tumor-free)	9 (52.9)
	Partial remission	4 (23.5)
	Recurrence	1 (5.9)
	Other	2 (11.8)
	Missing	1 (5.9)
**Treatment status, n (%)**		
	Ongoing	14 (82.4)
	Completed	2 (11.8)
	Missing	1 (5.9)
**Previous treatment^a^, n (%)**		
	Surgery	12 (70.6)
	Radiotherapy	7 (41.2)
	Chemotherapy	11 (64.7)
	Immunotherapy	2 (11.8)
	Antihormone therapy	1 (5.9)
	Antibody therapy	1 (5.9)
**Treatment location^a^, n (%)**		
	University clinic	17 (100)
	Outpatient practice	3 (17.6)
**Time since diagnosis (months)**		
	Mean (SD)	40.3 (52.0)
	Range	4-199

^a^Multiple answers possible.

### Interview Results

In total, we coded 1162 text passages and assigned them to the deductively formed main categories. Inductively, subcategories (n=44) were formed and specifications were made up to the fourth sublevel. Since the aim of the study was to derive implications for app development from the interviews, we focused on the results with respect to further development of the MUC app. Table 3 shows a summary of the category system.

**Table 3:** Summary of the category system.

Main category	Subcategory
Benefits of the MUC^a^ app	Time savings for patients and medical staff Paper savings COVID-19–conditioned contact reduction
Concerns about the MUC app	Data security and confidentiality Replacement of personal contact with the practitioner Concerned by negative information Loss of control Too much information Hidden costs
Requested app features	Support for a healthy lifestyle List of contact persons Networking with other institutions Organizational matters Social service themes Medication management Support for coping with the disease Exchange with others Feature for relatives Audio recording of the doctor’s appointment
Comments on basic feature 1 (appointment display and navigation)	From *remarks on app structure*: Possibility to book and manage appointments Arrival tips Preparation for treatment appointments Indication of waiting times and examination duration Location plan Link to Google Maps
Comments on basic feature 2 (access to medical reports)	From *remarks on app structure*: Central overview of medical reports Data transmission between general practitioner and hospital Processing status of cancer finding
Comments on basic feature 3 (forms and questionnaires)	From *remarks on app structure*: Control by doctors Fill-in help
Comments on basic feature 4 (health diary)	From *remarks on app structure*: Resulting consequences Limitation to specific aspects Feedback on health status Image transmission in the case of suspicion (skin cancer)
Comments on basic feature 5 (information about the clinic and the disease)	From *remarks on app structure*: General information (eg, digitization of flyers, individualization of information) Information about treatment (eg, description of treatment options and consequences, treatment process) Information about the disease (eg, disease stages, genetic testing of children)
Aspects for optimizing acceptance and usability	Technical aspects (eg, reminder function, selection and deselection of features) Design aspects (eg, clear structure, absence of advertisements) Communication about the MUC app (eg, recommendation by doctors, active thematization of data protection) Patient characteristics to facilitate app usage (eg, young age, chronic disease)

^a^MUC: My University Clinic.

#### Perceived Benefits of the MUC App

Patients mentioned some general *benefits of the MUC app*. These benefits included time savings for patients and medical staff (n=11, 64.7%), as well as paper saving (n=8, 47.1%) with respect to the goal that digitalization could replace printouts. Concerning *access to medical reports*, some patients had a positive view regarding always having medical records digitally available in order to check certain details (n=12, 70.6%). This aspect was assessed as being particularly important when patients change from primary care to outpatient care with private practice physicians (n=9, 52.9%). Faster transmission of medical reports via the MUC app was another perceived benefit (n=4, 23.5%). The possibility to fill in *forms and questionnaires* in the MUC app appealed to patients as it might be a facilitation for the clinic staff, where patients have more time to fill in forms (n=5, 29.4%) and are able to look up necessary medical details (n=4, 23.5%). Patients rated that a *health diary* could help them feel more confident (n=5, 29.4%) and better prepared for their medical consultations (n=2, 11.8%). Entries in a health diary might be more reliable than what patients remember from memory (n=4, 23.5%). Many patients favored the function *information about the disease* (eg, the statement that more information might reduce their anxiety; n=5, 29.4%).

So I imagine that you simply take away fears through educational information. Because fears are also caused by ignorance.

Interview 11, item 78

In addition, most patients perceived the MUC app as a trustworthy source of information (n=11, 64.7%).

I mean, I can be sure that when the University Medical Center provides information about my illness, that it is correct.

Interview 15, item 196

### Perceived Concerns About the MUC App

Data security and confidentiality were the most frequently mentioned *concern*s. Therefore, patients suggested that the MUC app should be password-protected (n=3, 17.6%), data transmission should be encrypted (n=2, 11.8%), and data should be deleted after a certain time (n=1, 5.9%). Other patients were not concerned about data security and confidentiality and expressed their trust in the MUC app (n=8, 47.1%). A further major concern was that personal contact with the physician could be reduced or replaced by the MUC app (n=9, 52.9%).

Well, it [the MUC app] should definitely not replace the doctor's consultation. I wouldn't like that.

Interview 12, item 121

The concern of losing control over the MUC app was important as well (n=7, 41.2%). To prevent this, patients wanted to be able to activate and deactivate individual functions (n=3, 17.6%). Patients also wanted the use of the MUC app to be voluntary at any time, with explicit consent being required for use (n=5, 29.4%). This aspect was particularly important for the function that allows the transfer of personal medical data to other medical staff or institutions (n=3, 17.6%). Patients pointed out that personal medical data should not be transferred to any other third parties, such as health insurance companies or banks (n=8, 47.1%). Concerning *access to medical reports*, patients expressed the concern that they might not be able to understand technical terms or may misinterpret or misunderstand the medical reports (n=5, 29.4%). Regarding a *health diary*, some patients were worried that the questions might contain intimate details of their lifestyle (n=3, 17.6%). Regarding the function *information about the disease*, some patients were concerned that they might be confronted with upsetting information about their diagnosis (n=8, 47.1%).

### Prerequisites for the Use of MUC App Features

All the patients in this study expressed the will to try and use the MUC app, even though some patients expressed the condition that all information should still be available in analogue format (n=5, 29.4%). Some patients expressed prerequisites for the use of the designed MUC app features (eg, some patients only wanted *access to their medical reports* via the MUC app, in combination with a face-to-face conversation with their doctor; n=2, 11.8%). In addition, 16 (94.1%) patients consented that their data may be transmitted via the MUC app to their general practitioner, but some patients wished a mandatory patient consent for this function (n=4, 23.5%).

### Suggestions for Improving the MUC App Features

Patients generated various ideas on how the individual basic features should be designed. They requested that the *appointment management and navigation* feature have a location map or route description to the appointment (n=2, 11.8%) and a link to Google Maps (n=1, 5.9%). In addition, some patients wished a checklist of required documents for a medical consultation (n=4, 23.5%) and an opportunity to take notes about medical examinations, a list of relevant questions to the doctor for the next appointment, and a general note function (n=3, 17.6%). Some patients wanted that *access to medical reports* be designed, including the possibility to send medical reports from the general practitioner to the Medical Center – University of Freiburg (n=6, 35.3%). This could minimize the frequent loss of information due to transmission via fax machines.

Then I had to go back to the general practitioner to find out where it [the blood count] was, why it wasn't faxed [to the clinic]. So, if you could maybe somehow solve this a bit differently or via the app.

Interview 10, item 42

For a better overview, some patients wished for an archive of all their medical reports concerning their cancer diagnosis (n=12, 70.6%). They also wanted automatic access to all medical records of the clinic and of their general practitioner, for example, diagnostic imaging (n=7, 41.2%).

Concerning the feature *forms and questionnaires*, patients requested fill-in help for forms in the MUC app (n=1, 5.9%) and that doctors should control the patients’ input for omissions and false data (n=4, 23.5%). Regarding the *health diary*, some patients requested feedback concerning their health status (n=4, 23.5%) and expected a response on what to do if the health state deteriorates (eg, being called in earlier for a check-up; n=2, 11.8%).

Concerning *information about the clinic and the* *disease*, the interviews revealed that the need for information about cancer was broad and contained general information, information about the disease, and information about treatment. Many patients wished for individualized information about their diagnosis (n=9, 52.9%) and also requested digitalization of flyers (n=9, 52.9%), links to serious websites (n=3, 17.6%), and a glossary of medical terms (n=5, 29.4%). Moreover, some patients requested information about new treatment methods or the possibility to participate in studies and new research findings (n=5, 29.4%). Many patients wished to get an overview on the clinics’ cancer-related supplementary health care programs (n=8, 47.1%), testimonials of other patients (n=3, 17.6%), and a description of treatment options and their consequences (n=6, 35.3%).

### Demand for Additional App Features

In addition to the 5 designed features of the MUC app, patients suggested a large number of additional *app features.* Some of the features listed next could be integrated into existing features. Patients asked for a list of health care professionals (n=5, 29.4%) with a direct function to contact them and ask medical questions (n=11, 64.7%). They also wanted it to be possible to communicate asynchronously (n=7, 41.2%).

But I think that now that we have come back to the contact persons and somehow the team to which I am now assigned, or to which I may turn. That is, I think, quite – quite nice to still have that somehow.

Interview 8, item 157

Most patients suggested app features to support healthy lifestyles (eg, advice on cancer-specific nutrition, suggestions for exercise and relaxation; n=16, 94.1%), as well as features to connect with other internal (eg, psychosocial counseling, sport oncology) and external (eg, cancer support groups, gene laboratory) services (n=9, 52.9%). Many patients named organizational matters (eg, daily schedule for inpatients and an overview of clinic departments to be easily depicted in the MUC app; n=12, 37.3%). They proposed to include topics of social security (eg, how to apply for the severely disabled status; n=9, 52.9%). Some patients wanted an app function with practical recommendations on how to cope with their disease (eg, by positive reports of how other patients with cancer successfully adapted to or overcame their disease; n=7, 41.2%). Patients also wished to have medication management in the MUC app (n=8, 47.1%). This function could include a reminder of when to take which medication and an explanation of the purpose of the medication.

### Improving Acceptance and Usability

Regarding aspects that increase the acceptance and usability of the MUC app, 4 themes emerged: a clear structure (n=10, 58.8%), easy handling (n=4, 23.5%), no advertising (n=2, 11.8%), and an appealing design (n=1, 5.9%). In addition, patients named technical aspects, such as a reminder feature that helps remember medical appointments, medication intake, or health diary entries (n=11, 64.7%). A few patients stated that they should be able to choose which features they want to use (n=10, 58.8%).

Yes, of course I have to be able to adapt it [the MUC app] to my needs without being a programmer. And that should to be different modules that I can then compose myself.

Interview 3, item 170

Some patients expressed their wish for the MUC app to be barrier free in terms of varying font sizes, voice control, or provision in other languages (n=9, 52.9%).

## Discussion

### Principal Findings

As far as we know, this study is one of the first to explore patients’ preferences and evaluate an app to support cancer treatment with a qualitative approach during its development in Germany. Our objective was to assess the needs and preferences of patients with cancer related to the MUC app and to identify possible barriers and concerns that might limit the acceptance of the app. Based on purposive sampling, we included 17 patients with cancer, reaching a satisfying variety of sampling characteristics. With 17 semistructured interviews, a saturation of the thematic content was reached. A key finding of our study is that all patients interviewed would test and use the MUC app. This indicates a high level of acceptance of the MUC app by patients, even if the benefits of the 5 app functions designed are judged differently.

Patients mentioned a variety of general benefits of the MUC app; the most common were time saving, less paperwork, and more rapid access to information [27]. The most important concerns were the fear that the MUC app might reduce personal contact with medical staff, and data security and confidentiality. On the one hand, there was the patients' desire for individualized information; on the other hand was their wish for privacy protection. We identified the patients' wish to restrict access to their individual health information and the worry that individual information could be compromised by third parties (eg, health insurance). In our study, we found 2 additional aspects: First, there was a clear statement that the use of the MUC app should be voluntary and not replace other analogue sources of information. Second, when using the MUC app, the user should be able to select features and deactivate those they do not want to use. We found that the MUC app might improve the flow of information between general practitioner and clinic and vice versa. It turned out that the health diary is a well-accepted and helpful tool, especially for patients during cancer treatment. Thereby, symptom monitoring can contribute to better health care [28,29] and to a feeling of safety for outpatients [19,30,31]. We detected a great variety of information needs in our study sample, which may reflect the heterogeneous sample characteristics (eg, the broad range of time since diagnosis, various treatment settings, ongoing and completed treatment).

As known from the literature, the need for information in patients with cancer is high [32-34] and changes over the course of cancer care [14,32,33,35,36]. It is important to meet these information needs, as access to health information has a significant effect on reducing anxiety and depression [37,38]. In our study, patients reported the wish for individualized information about diagnosis and treatment to reduce anxiety. In practice, it is important that an app provide individualized information, depending on the stage of treatment, as this is seen as an essential requirement for the successful use of app-based assistance. Most patients in our study stated that they would estimate the MUC app as a trustworthy source of information. Nevertheless, with respect to information, we found both advantages, such as a reduction in anxiety, and disadvantages, such as being concerned by too detailed information [11]. As a possible explanation, the perception of information may be influenced by different coping strategies: patients predominantly using an avoidant coping strategy [39-41] may be more afraid of detailed information. In addition, according to the common-sense model (CSM) [42], cognitive factors, such as information from the external social environment (eg, caregivers or authoritative sources, such as physicians), influence illness representations, illness coping behaviors, and illness and emotional outcomes.

Beyond the designed app features, patients suggested some new app features, such as an interactive contact list and support for a healthy lifestyle. These aspects have also been identified as patients' needs in other studies [14,43]. In line with previous studies are concerns that the MUC app may reduce face-to-face contact with health care professionals [13,19], as well as concerns about data security and confidentiality [14,44-46]. Consistent with existing studies, patients want control over who has access to their personal health information [47,48]. Concerns that personal information will be interpreted to the patient's disadvantage (eg, by a health insurance company) are also consistent with existing evidence [47]. As found in the literature [49], patients requested an interactive communication feature in mHealth tools instead of a unidirectional delivery of information. This emphasizes the potential benefit of bidirectional communication between patients and physicians in the MUC app. Asynchronous communication with medical staff was another important desire identified in our study. The transmission of data (eg, between the outpatient and inpatient health care sector) via the MUC app was partly seen as useful. These aspects could help improve trans-sectoral communication and optimize patient-centered care.

Young age [11,13,50] and open-mindedness toward mHealth [47] was found both in the literature and in our study to be a common patient characteristic to facilitate app usage. A new finding from our study is that patients explicitly wish for medication management via the MUC app. Our study provides evidence that medication management is seen as an important part of an app by patients with cancer. In addition, as far as we know, it has not yet been documented in the literature whether the voluntary use of the MUC app and the possibility to decide individually which functions should be used are relevant aspects from the patient's point of view. Both aspects could be linked to the desire for more patient autonomy and should definitely be considered in app development.

### Limitations

There are a number of limitations to our research. Although we used purposive sampling, it is possible that patients with low technical skills were either not approached by physicians to participate in the study or did not feel interested by the study call. It is also possible that mostly patients with open-mindedness toward mHealth took part in our study. This may have led to participants being more positive about mHealth than the overall population of patients with cancer. There might have been a response bias in the direction of social desirability, as the interviewer was probably seen by the patients as a clinical representative. As a monocenter study, the generalization of our results in terms of mHealth apps in general is not possible, as some topics named by the patients may be specific to their oncological care situation at the Medical Center – University of Freiburg. Furthermore, patients were asked to imagine a hypothetical app they might use during their illness. Even though the functions of the app were explained in detail and clearly by using visual material, the data are not based on concrete experiences with the MUC app itself. Consequently, expressing a desire for a particular app function does not automatically imply that that person will use the app function as soon as it is available.

### Conclusion

The patients’ wishes and concerns revealed early in the development process show the relevance of involving patients in the development of mHealth apps. During app development, it should be kept in mind that patients with cancer are more often older patients, which means that the app should be clear and simple in structure. In addition to technical aspects, communication about the app is important, which is why possible concerns about data privacy should be actively thematized. The COVID-19 pandemic may increase the acceptance and need for mHealth apps to support contact-free health care [51].

The findings provide insights into how to improve the MUC app based on the patients’ perspective. The study reported in this paper comprises a second phase, in which patients will receive a test version of the app. At the end of the test phase, interviews will be conducted to gather feedback and suggestions for improvement. It seems important that the MUC app should not reduce but optimize personal contact with health care professionals. The MUC app may contribute to the improvement of the relationship between practitioner and patient by simplifying organizational processes. Implementation of the MUC app requires education by clinic staff for those patients with low technical experience [44,52]. Patients and patients’ representatives should be involved in all subsequent phases of app development.

## Appendix

Multimedia Appendix 1 Interview guide.

## Abbreviations

CCCF: Comprehensive Cancer Center Freiburg
mHealth: mobile health
MUC: My University Clinic
PROM: patient-reported outcome measure

## Data Availability

The qualitative data are available from the corresponding author upon reasonable request.
