# Supplementary material for: Identification of the Needs and Preferences of Patients With Cancer for the Development of a Clinic App: Qualitative Study
Source: JMIR Cancer. 2023 Jul 27;9:e40891. doi: 10.2196/40891 (PMC10415940; doi:10.2196/40891)
Supplement: Multimedia Appendix 1 [file cancer_v9i1e40891_app1.pdf]

UNIVERSITÄTS KLINIKUM FREIBURG
Stiftungsprofessur
Selbsthilfeforschung
CCC Freiburg

Interview guide for needs assessment: individual interview without prior app use

Introduction

My name is …, I work here in the Department of Self-Help Research. First of all. Thank you for taking the time for this interview. You have already received the patient information from us. As was described in the information, a patient app for oncology care is being developed here at the University Medical Center Freiburg. In this interview, I would like to ask you about your opinion regarding such an app and whether you have any specific wishes or fears about such an app. This interview should help to include the perspective of patients in the development of the app and to design the app in such a way that future users will be satisfied with the app.

The interview actually runs like a normal conversation. I will ask various questions and you simply tell me everything you think is important about this question. We are interested in your personal opinion and experience on this topic. The interview will last about 30-45 minutes.

A brief word about the formalities: As already mentioned in the patient information, I would like to make an audio recording of the interview for later evaluation. I use this audio recorder for this purpose. The audio recording also helps me to follow the interview better, because then I do not have to write everything down. Of course, we will treat the material from the interview strictly confidentially and the evaluation will only take place pseudonymized, i.e. encrypted.

Is that okay with you?

Good, do you have any questions about the interview or the study?

[Read through consent form and have it signed]

[Turn on voice recorder]

Interview

General attitude towards apps		
**Introductory question:**<br>**First of all, I am interested in your experience with apps so far?**		
**Content topics**	**Key questions**	**Steering questions**
Experience with apps	Do you use a smartphone in your everyday life?<br><br>Do you use apps?<br><br>Which apps do you use?<br><br>How often do you use apps?	*What do you know about apps? (if it is not clear what apps are)*<br><br>*Could you describe this in more detail/ in greater detail?*<br><br>*Why don't you tell us a little bit more about it?*
Attitude towards apps	What do you think of apps in general?	*Can you give an example of ...?*<br>*Can you think of anything else about this?*<br><br>*Is there anything missing, or can we move on?*

General attitude towards apps in healthcare		
**Introductory question:**<br>**There is an increasing number of apps that are being used in healthcare. Have you ever heard of them?**		
**Content topics**	**Key questions**	**Steering questions**
Own experience	Have you perhaps had any experience with such apps in the health sector?	*What do you think about it?*<br><br>*And what else?*
Experiences of others	If no: Do you know anyone who has had experience with this?	*Can you think of anything else to say about this?*<br><br>*Is there anything missing, or can we move on?*<br><br>➔ *Is this person using it? Do you go there for advice?*

**Needs assessment: Apps in oncology care**		
**Introductory question:**<br>**Have you ever thought about an app to support you during cancer treatment?**		
**Content topics**	**Key questions**	**Steering questions**
Needs assessment based on care pathway	**When you think of the following situations, what support would you have liked/ could you imagine needing and how could this be covered by an app?**	
**The time before your treatment began:**<br>expectations	If you think of the time before your treatment began, what would you expect from such an app?	
Wishes for Functions	What should such an app be able to do? /What capabilities should the app offer you?	*What would be helpful for you?*
Concerns	What concerns or fears would you have?<br>Do you see any disadvantages?	*What do you not want?*
**The time during treatment:**<br>expectations	When you think about the time during treatment, what would you expect from such an app?	
Wishes for Functions	What should such an app be able to do? /What capabilities should the app offer you?	*What would be helpful for you?*
Concerns	What concerns or fears would you have?<br>Do you see any disadvantages?	*What do you not want?*
**The time after the end of treatment:**<br>expectations	When you think about the time after the end of treatment, what would you expect from such an app?	

Wishes for Functions<br><br><br><br>Concerns	What should such an app be able to do? /What capabilities should the app offer you?<br><br>What concerns or fears would you have?<br>Do you see any disadvantages?	*What would be helpful for you?*<br><br><br><br>*What do you not want?*
Acceptance of an app in oncology care	Would you use such an app if the University Medical Center Freiburg would offer it?	*What would be important to you?*<br>*What should be taken into account?*

**Barriers**		
**Introductory question:**<br>**What aspects might prevent you from using the app?**		
**Content topics**	**Key questions**	**Steering questions**
Barriers	What would make it difficult for you to use the app? (Design, end devices, handling, etc.)<br><br>What aspects would make it easier for you to use the app?	*Could you describe this in detail?*<br><br>*Why don't you tell a little bit more about it?*<br><br>*Can you give an example of ...?*<br><br>*Can you think of anything else about this?*

Suggestions for improvements in the University Medical Center Freiburg		
**Introductory question:<br>Thinking about your experience with University Medical Center Freiburg so far, how satisfied are you overall with the care situation?**		
**Content topics**	**Key questions**	**Steering questions**
Improvement of processes	Where are procedures not yet ideal in your eyes?<br><br>Where could an app improve things?	*Could you describe this in more detail?*<br><br>*Why don't you tell a little bit more about it?*<br><br>*Can you give an example of ...?*<br><br>*Can you think of anything else about this?*

Opinion on planned app features		
**Introductory question:<br>I would like to introduce you to what the app from University Medical Center Freiburg will probably be able to do and ask you to tell me what you think of it:**		
**Content topics**	**Key questions**	**Steering questions**
Appointment module with appointment reminder and navigation to the appointment<br><br>Access to medical reports via the app (medication plans, images, blood counts, doctor's letters, findings)	What do you think of this feature?<br>Which advantages and disadvantages do you see in it?<br>Would you use it? Why (not)?<br><br>What do you think about this feature?<br>Would you use it? Why (not)?<br><br>Could you imagine your health data, such as medical reports, being transmitted to third parties via the app? What do you think about this? Which	*And what else?*<br><br>*What other advantages and disadvantages do you see in it?*<br><br>*Can you think of anything else to add?*<br><br>*Is there anything else missing, or can we move on?*

	advantages and disadvantages do you see?	
Forms and questionnaires (registration forms and health questionnaires)	What do you think about this feature?<br>Which advantages and disadvantages do you see in it?<br>Would you use it? Why (not)?	
Health diary (health status, e.g., blood pressure; well-being, e.g. pain; lifestyle, e.g. diet, sleep) for submission to practitioners	What do you think about this feature?<br>Which advantages and disadvantages do you see in it?<br>Would you use it? Why (not)?	*Which other advantages and disadvantages do you see in it?*
Information about the University Medical Center Freiburg and the disease	What do you think about this feature?<br>Which advantages and disadvantages do you see in it?<br>Would you use it? Why (not)?<br><br>How well do you feel informed about your disease and additional services offered by the University Medical Center Freiburg (e.g. sports with cancer, lectures, self-help groups)?<br>If you do not feel well informed so far, what other sources do you use to fill this gap?	*What other advantages and disadvantages do you see in it?*

Opinion on other possible app features		
**Introductory question:**<br>**It is quite conceivable that the app could offer other features as well. Can you think of anything else that would be helpful for you?**		
**Content topics**	**Key questions**	**Steering questions**
Domain: Information	When you think about the area of information, can you think of something that you could use/ where the app could help you?	*(→ Input: links to reliable websites, event information about cancer, etc.)*<br>*What do you think about this? Would you use something like this? Why (not)?*
Domain: Functions in case of deterioration	Is there anything that would be helpful to you when you're feeling down?<br>Could you use a platform where you can enter something?	*(→ Input: contact function in case of symptom worsening, contact addresses, graphical overview of symptom diary entries)*<br>*What do you think about this? Would you use something like this? Why (not)?*
Domain: Self-care	If you think about the area of "assistance in everyday life", i.e. also what you can do for yourself, do you have any ideas about how an app could support you there?	*(→ Input: physical, i.e. nutrition, exercise, wound care; psychological, i.e. relaxation, exercise)*<br>*What do you think about this? Would you use something like this? Why (not)?*

Conclusion

From my side, I have no more questions for you. Is there perhaps something you would still like to address, perhaps something we did not discuss or something that came up a little short in the conversation? Perhaps you also have another concern or question?
Then I **thank you** very much for your time and the conversation!

[Switch off dictation machine]
